# Exploring the Immunological Landscape of Ischemia/Reperfusion Injury and Graft Rejection in Kidney Transplantation: Shared Mechanisms and Insights

**DOI:** 10.3390/cells14181443

**Published:** 2025-09-15

**Authors:** Dario Troise, Barbara Infante, Silvia Mercuri, Bengt Lindholm, Karolina Kublickiene, Giovanni Stallone

**Affiliations:** 1 Nephrology, Dialysis and Transplantation Unit, Advanced Research Center on Kidney Aging (A.R.cK.A), Department of Medical and Surgical Sciences, University of Foggia, 71122 Foggia, Italy; barbara.infante@unifg.it (B.I.); silvia.mercuri1990@gmail.com (S.M.); giovanni.stallone@unifg.it (G.S.); 2 Renal Medicine and Baxter Novum, Department of Clinical Science, Intervention and Technology, Karolinska Institutet, 141 52 Stockholm, Sweden; bengt.lindholm@ki.se (B.L.); karolina.kublickiene@ki.se (K.K.)

**Keywords:** kidney transplantation, ischemia/reperfusion injury, rejection, delayed graft function

## Abstract

Background: Ischemia/reperfusion injury (IRI) is considered one of the major risk factors involved in the development of delayed graft function that significantly impacts both the early and long-term function of allografts due to its harmful effects on cells. Purpose: This narrative review aims to explore the emerging aspects of IRI in organ transplantation, focusing on the still unclear relationships between IRI and the development of both T-cell-mediated and/or antibody-mediated rejections. Key findings: Recently, efforts aimed at increasing the knowledge of the mechanisms involved have revealed that IRI is connected to rejection processes through a complex of interconnected pathways. These pathways affect both the viability and the metabolism of immune cells, ultimately influencing graft outcomes. Therefore, these pathways demonstrate the complexity of immune responses after transplantation and play a role in both acute and chronic rejection processes. Conclusions: Improving graft outcomes requires an understanding of the multifaceted relationship between IRI and immune-mediated rejection, which is critical to improve graft outcomes. Further research is needed to clarify these mechanisms and develop targeted strategies to mitigate IRI and its impact on transplant rejection.

## 1. Introduction

Organ transplantation serves as the gold standard therapy for patients suffering from organ failure [1]. According to the European Commission, kidneys are the most frequently transplanted organ, followed by liver, heart, and lungs; thus, there is an expanding worldwide demand for organ transplantation [2]. One of the main factors that could influence outcomes and prognosis, and potentially may limit the success of the transplantation, is cell damage. Ischemia/reperfusion injury (IRI) can cause cellular damage in transplanted organs [3].

IRI is defined as a paradoxical increase in cellular dysfunction and death that occurs when blood flow to previously ischemic tissues is restored [4]. IRI is also considered a major risk factor involved in the development of delayed graft function (DGF), mostly defined as an acute kidney injury (AKI) that occurs in the first week after transplantation, requiring dialytic treatment. DGF is a common complication, occurring at rates of 25% to 30%, and represents one of the most challenging clinical problems for kidney transplant recipients due to its association with poor short- and long-term outcomes and higher rejection rates [5]. Nevertheless, studies have underlined that the real consequence of DGF on kidney outcomes is not yet completely understood. Several studies highlighted the detrimental effects of DGF on long-term graft survival, while others suggest that it has a negative impact only during the first year post-transplantation, and still others have found no significant effects. In a meta-analysis, Li et al. showed a higher incidence of acute rejection in DGF patients versus those who did not experience DGF [6]. Moreover, acute rejection can trigger and lead to chronic kidney rejection. Thus, IRI may increase the risk of developing chronic rejection during the long-term post-transplantation period [7]. Chronic rejection is a leading cause of kidney transplant failure, representing a common manifestation of a sustained alloimmune response. It can manifest, as proposed in the recent Banff classification, as chronic (active) antibody-mediated rejection (AMR) and chronic T-cell-mediated rejection (TCMR). Progressive and irreversible nephron loss and subsequent decline in renal function are considered pivotal factors in the development of chronic rejection [8].

The scientific problem of how IRI contributes to acute and chronic rejection is still not fully understood, despite advancements in immunosuppressive techniques and preservation techniques. Understanding this problem is critical for developing interventions to improve graft survival and function. In particular, clarifying the role of specific immune subsets and identifying effective therapeutic targets are key areas requiring further research. The aim of this narrative review of ischemia/reperfusion injury and its role in transplant rejection is to explore the interconnected pathways between IRI and kidney rejection, and potential therapeutic strategies to mitigate damage.

## 2. The Role of Immunometabolism in IRI and Allograft Rejection

Metabolism influences immune cells, and changes in immunometabolism can regulate cell development, function, and differentiation, thereby influencing the balance between pro-inflammatory and regulatory cells, ultimately impacting organ transplant outcomes. Changes in immunometabolism during IRI and allograft rejection share some features, and a better understanding of metabolic pathways in immune cells could modulate alloimmune responses, shifting specific immune cell subsets toward more tolerogenic profiles [9].

### 2.1. Cellular Changes During Ischemia/Reperfusion Injury

Ischemia resulting in severe tissue hypoxia leads to cellular metabolic changes. Afterwards, the reperfusion phase leads to further damage, which causes increased production of reactive oxygen species (ROS), calcium overload, inflammation, and activation of several pathological pathways involving immune and endothelial cells (ECs), resulting in decreased function and viability of the graft [10,11,12].

On a cellular level, a hypoxic environment causes a metabolic switch from aerobic respiration to anaerobic glycolysis, induced by ischemia and followed by a subsequent depletion of adenosine triphosphate (ATP). This is considered the “energy currency” of the cells because it provides quickly available energy to fuel cellular functions. Moreover, anaerobic glycolysis results in lactate production linked to a significant increase in protons, which may reduce intracellular pH via activation of Na^+^-H^+^ exchange and massive influx of Na^+^. Consequently, compensation for the disruption in ionic homeostasis results in intracellular Ca^2+^ overload through the plasmalemmal Na^+^-Ca^2+^ exchanger. It has been shown that the increased Na^+^ influx may be attributed to a second possible mechanism following the metabolic switch to anaerobic glycolysis induced by hypoxia. This shift is associated with increased entry of Na^+^ and glucose into the cytosol via the Na^+^-glucose cotransporter, which causes the activation of the Na^+^-Ca^2+^ exchanger and subsequent Ca^2+^ overload. Moreover, another mechanism has been hypothesized. Under a hypoxic environment, ATP depletion decreases the activity of the Na^+^-K^+^-ATPase pump, leading to increased intracellular Na^+^. These studies have demonstrated that Na^+^ influx has a crucial role in the increase in intracellular Ca^2+^, regardless of the underlying mechanism [13,14,15]. Calcium-dependent protein hydrolases (such as calpain) and proteases (such as caspases), as well as endonucleases and phospholipases, can be activated due to calcium overload, leading to cell damage and death [16,17]. Notably, high levels of intracellular calcium compromise mitochondria structure and damage outer and inner mitochondria membranes, leading to the collapse of the energy homeostasis and ATP consumption, in the futile attempt to restore homeostasis. During this phase, mitochondria are transformed from ATP-producing to ATP-consuming organelles. Furthermore, the opening of mitochondrial permeability transition pores (mPTPs) at the inner mitochondrial membrane causes the release of large amounts of Ca^2+^ and proapoptotic proteins from mitochondria, subsequently leading to cell death [18]. However, the contribution of mPTPs to cell death is not the same across all organs. While it represents a central mechanism in many tissues, such as the heart and brain, comparative studies have shown that kidney mitochondria demonstrated a more rapid and pronounced mPTP opening than brain mitochondria, suggesting organ-specific variability in this process [19,20]. In the early stages of ischemia, all mitochondria show shrinkage, condensation of the matrix, and widened cristae. After approximately 60 min, some mitochondria begin to swell, which reduces ROS clearance, ultimately leading to oxidative stress [21,22]. In addition to these mechanisms, the particularities of cold ischemia during organ preservation deserve attention because they represent a distinct form of mitochondrial injury. While hypothermia reduces metabolic demand, it does not completely prevent ATP depletion. Early studies on apoptosis following cold ischemia showed that in human proximal tubular epithelial renal cells, cold storage can induce mitochondrial swelling within 2 h because of the increased opening of mPTPs [23]. Subsequent research demonstrated that cold storage during organ preservation reduced mitochondrial complex I, II, and III activities and disrupted essential processes of mitochondrial fusion and fission. Additionally, cold ischemia also reduces ATP-synthase activity, thereby disrupting mitochondrial energy homeostasis [24].

**Figure 1:**
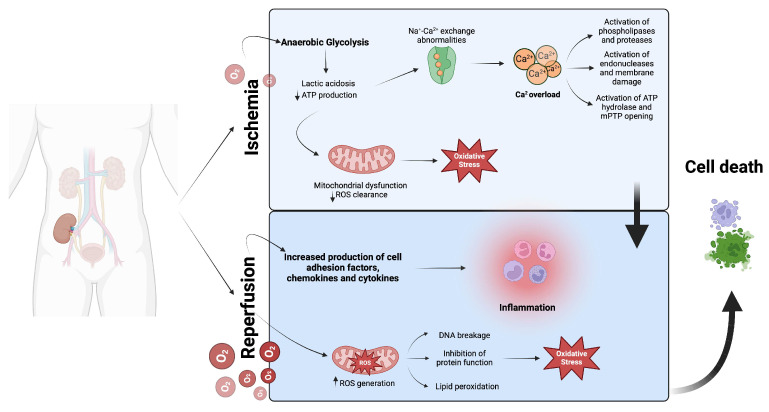
Ischemia/reperfusion injury causes cellular damage. Ischemia/reperfusion injury induces cellular damage through hypoxia-driven metabolic shifts leading to acidosis, ATP depletion, ionic imbalance, and mitochondrial dysfunction. Reperfusion amplifies injury via excessive ROS generation, inflammation, and mPTP opening, resulting in oxidative stress, biomolecular damage, and cell death. Abbreviations: mPTP, mitochondrial permeability transition pore. Created in BioRender. Troise, D. (2025) https://BioRender.com/19jb9sk [accessed on 1 September 2025].

The increased production of cell adhesion factors, chemokines, and cytokines, as well as the oxidation of succinate, previously accumulated in the ischemic tissue, aggravates tissue damage during the reperfusion phase. Indeed, succinate is rapidly oxidized by succinate dehydrogenase, resulting in a burst of ROS production by mitochondrial complex I. This triggers lipid peroxidation, DNA breakage, and inhibition of protein functions, further exacerbating oxidative stress [25,26] (Figure 1).

### 2.2. Metabolic Reprogramming of Immune Cells

The exploration of the metabolic network of innate and adaptive immune system cells is of crucial importance in the field of transplant immunology for understanding the mechanisms of organ rejection and developing strategies to prevent allograft injury. Cellular metabolism has been shown to play a critical role not only in immune cell survival and function but also in regulating immune cell signaling pathways. Under normal oxygen conditions, mitochondrial oxidative phosphorylation (OXPHOS) serves as the main metabolic pathway in naïve or resting T-cells to ensure the energy needs for immune surveillance. In contrast, both CD4^+^ and CD8^+^ effector T-cells switch to aerobic glycolysis to meet their biosynthetic demands [27]. This metabolic shift, known as the Warburg Effect, was first observed by Otto Warburg (1920) in cancer cells and later recognized as important in activated T-cells. Jacobs et al. demonstrated that the upregulation of glucose transporter GLUT1 on the surface of T-cells is critical for T-cell activation and accumulation of activated memory T-cells. In contrast, GLUT1 expression is reduced in naïve T-cells [28]. Moreover, the uptake and metabolism of amino acids, especially glutamine, are required for T-cell function. Carr et al. showed that depletion of glutamine results in inhibition of cell proliferation and cytokine production [29]. Interestingly, T-cell activation requires a balance between glycolysis and OXPHOS, rather than an all-or-none response. Tan et al. reported that during the initial phase of T-cell activation, anaerobic glycolysis seems to be less important, while OXPHOS, although less upregulated than glycolysis, is essential for their activation [30]. Additionally, regulatory T-cells (Tregs), which play a pivotal role in downregulating allograft rejection and inducing immune tolerance to the allograft, have been shown to be affected by fatty acid oxidation (FAO) and OXPHOS rather than glycolysis, as demonstrated in vivo using GLUT-1-deficient mice [31,32].

Furthermore, T-cell activation heavily relies on the function of stimulatory donor- and recipient-derived dendritic cells (DCs). They play a pivotal role in both allograft tolerance and rejection by infiltrating the transplanted organ and sustaining T-cell-mediated responses [33]. Metabolic changes that affect DCs, after Toll-like receptors (TLRs) engagement, can be divided into two phases. The early phase is characterized by a glycolytic burst and a reduction in OXPHOS, which is suppressed by nitric oxide (NO) production from inducible NO synthase (iNOS). Consequently, in the late phase, the metabolic profile of DCs shows a decrease in aerobic glycolysis occurring after around 9 h from the activation of DCs through TLRs [34]. At this stage, it is also important to address the role of macrophages that contribute to the pathogenesis of IRI and to acute and chronic allograft rejection by secreting inflammatory mediators and enhancing adaptive immune responses, promoting tissue injury. Macrophage subsets are characterized by different metabolic profiles: M1-type macrophages, recognized for their pro-inflammatory phenotype, and M2-type macrophages, known for their anti-inflammatory properties. Under hypoxic conditions, as it happens during the ischemic phase of IRI, high levels of hypoxia-inducible factor 1α (HIF-1α), which promotes M1 differentiation and sustains glycolysis activity, were observed in the cytoplasm of human monocytes and macrophages. Indeed, M1-type macrophages predominantly rely on glycolysis for ATP generation, while M2-type macrophages show increased FAO and OXPHOS [35,36].

B-cells play a crucial role in late immune-mediated kidney injury. Activated B-cells, which produce donor-specific antibodies (DSAs) resulting in allograft rejection, were also found to be abundant in the kidney after IRI. B-cells can be divided into B1 B-cells, which contribute to innate immune responses, inflammation, and activation of neutrophils and macrophages, and B2 B-cells, involved in humoral immunity and chronic rejection-related damage [37]. Metabolic reprogramming has recently been well described in the context of naïve B-cell activation/differentiation and is essential for antibody production. Importantly, B-cell lineages may rely differently on glycolysis. For example, HIF-1α is essential for the expression of genes related to glycolysis and lipid metabolism in B1 B-cells, and HIF-1α-deficient mice showed abnormal peritoneal B1 B-cells associated with autoimmunity, probably due to the deficient expression of glucose transporters and glycolytic enzymes [38]. Additionally, B1 B-cells are bioenergetically more active than B2 B-cells, exhibiting higher rates of glycolysis, on which they depend, and increased OXPHOS [39]. However, it has been demonstrated that activated B-cells undergo different metabolic reprogramming responses compared to those that occur in T-cells. Initially, their activation involves increased metabolism without specific shifts in the balance between lactate production and oxygen consumption. Later, chronic exposure to B-cell-activating factors (BAFFs) leads to rapid induction of aerobic glycolysis and metabolic reprogramming, crucial for proliferation and antibody production [40]. Moreover, glucose was not required for the initial activation of B-cells, and instead, they showed enhanced OXPHOS, tricarboxylic acid (TCA) cycle activity, and nucleotide biosynthesis. This suggested that glucose restriction had a relatively minor impact on T-cell-dependent B-cell activation compared to the inhibition of OXPHOS or glutamine restriction [41].

## 3. Innate Immune Responses Involved in the Development of IRI and Rejection

### 3.1. Toll-like Receptors

Similar to AKI in native kidneys, DGF is associated with the activation of the innate immune system in transplanted grafts. The cellular damage that occurs after IRI provokes a massive release of compounds from the damaged tissue and cells, known as damage-associated molecular patterns (DAMPs), including hyaluronic acid, fibronectin, heat shock proteins, and high mobility group box 1 protein. TLRs, a class of pattern recognition receptors (PRRs), recognize DAMPs, and their activation is crucial for the initiation of innate immunity [42,43]. In the kidney, their expression has been reported in podocytes, tubular epithelial cells, and glomerular ECs, suggesting a role in the activation of immune responses in tubulointerstitial injury [44], glomerulonephritis [45], and vasculitis [46]. Several TLRs have been found in mammals. TLRs 1, 2, 4, 6, and 10 are located on the cell membrane surface, while TLRs 3, 7, 8, and 9 reside intracellularly [47]. Among the TLRs, TLR2 and TLR4 significantly influence the pathological development of IRI. Their activation promotes the release of pro-inflammatory mediators from leukocytes and other cells, the activation of the innate and adaptive immune systems, and the enhancement of renal fibrosis during IRI of the transplanted kidney [48,49]. Interestingly, antigen-presenting cells (APCs), including dendritic cells and macrophages, and tubular epithelial cells constitutively express TLR2 and TLR4, with this expression increasing during kidney IRI [50,51]. Loss-of-function TLR4 mutation in kidney parenchymal cells prevented the rise in pro-inflammatory cytokines and is associated with improved immediate allograft function in kidney IRI models [52]. Additionally, the absence of TLR2 protects mice from ischemic renal injury [53]. After being activated, TLRs elicit the production of pro-inflammatory cytokines and chemokines via the NF-kB pathway through the adapter protein MyD88, including interleukin 1 (IL-1), interleukin 6 (IL-6), tumor necrosis factor alpha (TNFα), and interferons (IFNs), leading to aseptic inflammation, migration of leukocytes, and activation of the immune system [54]. Except for TLR3, whose activation is mediated by the recruitment of TICAM-1 (TIR domain-containing adaptor molecule 1), TLRs signaling necessitates the recruitment of MYD88 [55].

**Figure 2:**
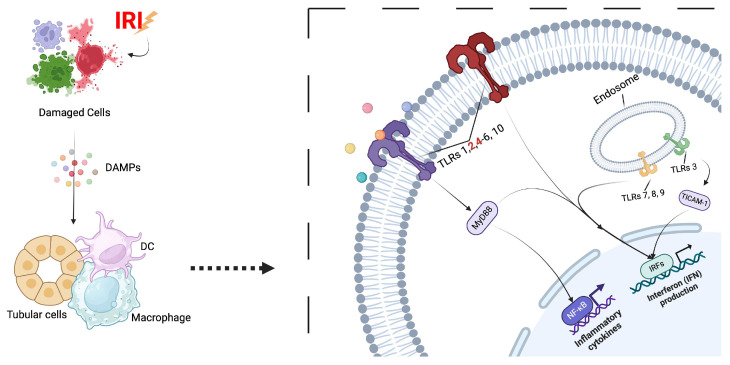
Toll-Like receptors activation during ischemia/reperfusion injury. Ischemia/reperfusion injury triggers the release of damage-associated molecular patterns (DAMPs), which activate Toll-like receptors (TLRs) on renal cells. TLR2 and TLR4 (highlighted in red), upregulated during injury, drive pro-inflammatory signaling and immune activation, contributing to further tissue damage and rejection. Except for TLR3, which recruits TICAM-1 for activation, TLR signaling generally requires the recruitment of MYD88. Created with BioRender.com. Troise, D. (2025) https://BioRender.com/86q1lnx [accessed on 1 September 2025].

In kidney transplanted patients, the roles of TLRs have also been implicated in allograft rejection. Findings have suggested that TLR4, implicated in the activation of the innate immunity system, contributes to the development of acute kidney rejection post-transplantation [56]. Additionally, loss-of-function mutations in TLR4 can alter signaling in response to high mobility group box 1 (HMGB1), reduce cytokine levels, and increase the rate of immediate graft function [57]. In liver transplant patients with acute rejection, TLR2 and TLR4 were found to be upregulated a week after transplantation, suggesting that TLRs could be used as potential biomarkers for organ rejection [58] (Figure 2).

### 3.2. Macrophages and Neutrophils

A massive influx of neutrophils and macrophages has been found approximately 30 min to one hour after reperfusion in the injured transplanted kidney, reaching a peak at 24 h and maintaining elevated levels for at least 6 days thereafter [59]. In response to DAMPs and pro-inflammatory mediators mainly released by T helper 1 (Th1) cells, neutrophils, and Natural Killer (NK) cells, such as TNFα and IFNs, infiltrating and resident monocytes may differentiate into M1-type macrophages, promoting the removal of damaged or deceased renal tubular epithelial cells and neutrophils but further amplifying IRI through the release of pro-inflammatory cytokines and ROS. Approximately 72 h later, T helper 2 (Th2) cells and Tregs are recruited to the injured renal tissue. These cells promote tissue regeneration, repair, and immunomodulation by secreting IL-4 and IL-13, resulting in a phenotypic shift into M2-type macrophages [60]. Additionally, macrophages are considered the predominant cell type that infiltrates the kidney allograft during rejection, and their presence is closely linked to both short- and long-term outcomes of the transplanted organ [61].

Rodent studies have suggested that within 24 h after transplantation, transcripts associated with macrophage activation were upregulated in kidney allografts [62], followed by a progressive increase in infiltrating macrophages. the processes can therefore contribute to kidney rejection through antigen presentation, co-stimulatory signals, cytokine production, and interaction with other immune cells [62].

In TCMR, macrophage infiltration is associated with M1 polarization as well as with increased cell proliferation. Studies demonstrated that the number of infiltrating macrophages not only correlated with the dialysis re-initiation but also with the severity of acute TCMR with arteritis, which is associated with increased infiltration of CD68^+^ cells, known as a marker of macrophages, in the peritubular and the perivascular compartments. Notably, the observation that CD68^+^ cells accounted for the majority of HLA-DR^+^ cells may suggest that macrophages developed increased antigen presentation capabilities. Differently, they showed that in AMR, macrophages exhibited mostly peritubular infiltration [63]. Moreover, depending on the cytokines secreted by macrophages, they can promote the differentiation of activated T-cells from an allograft-rejecting Th1/Th17 phenotype to an allograft-accepting forkhead box P3^+^ (FOXP3^+^) phenotype. It has been demonstrated that donor polymorphisms in the gene encoding signal regulatory protein α (SIRPα), a mediator of the “do not eat me” signal, modulate the innate immune response in mice lacking adaptive immune cells. The engagement of SIRPα by CD47 suppresses DC maturation and macrophage phagocytic function. Therefore, the activation of the CD47/SIRPα pathway in recipients who differ from the donors in one or both SIRPα alleles prompts an innate alloresponse, further suggesting that macrophages can recognize SIRPα polymorphisms and initiate the rejection response. In line, depleting macrophages using liposomal-clodronate or a cFMS kinase inhibitor improved graft function in mice with TCMR of the kidney graft [64,65], further strengthening that macrophage lineage has a pivotal role in cell-mediated rejection.

In chronic allograft rejection, fibrosis and vasculopathy are the main pathological features. Macrophages, particularly the M2 phenotype, play a crucial role in the development of these changes, and the production of TGF-β by M2-type macrophages is considered a key factor for the induction of fibrosis of the graft. Moreover, in human transplanted kidneys with chronic injury, macrophage–myofibroblast transition (MMT) has been observed, and M2-type macrophages were found to co-localize with α-smooth muscle actin (α-SMA) positive myofibroblasts in the areas of interstitial fibrosis [66,67,68].

Renal endothelial cells (RECs) play a pivotal role in the neutrophil infiltration during IRI. RECs express several intercellular adhesion molecules, including intercellular adhesion molecule-1 (ICAM-1) and E-selectin, as well as chemokines and other inflammatory modulators. RECs become activated during IRI and undergo phenotypic changes characterized by the increased expression of pro-adhesive molecules [69], which leads to neutrophil recruitment, adhesion, and migration. IRI has been found to increase neutrophil size, promote their clustering, and produce more elongated neutrophils, leading to enhanced neutrophil activation and production of ROS, pro-inflammatory cytokines, release of proteases, and the formation of neutrophil extracellular traps (NETs) [70]. Neutrophils are also crucial in the initiation of acute cellular rejection. El-Sawi et al. demonstrated that the inhibition of CXCR2, a neutrophil chemokine receptor, reduces neutrophil infiltration and suppresses T-cell infiltration in a mouse model of cardiac allograft rejection. Furthermore, combining costimulatory blockade with peri-transplant neutrophil depletion and/or anti-CXCL1/2 antibodies significantly prolongs cardiac allograft survival, suggesting that early neutrophil-induced tissue damage could facilitate T-cell-mediated rejection [71]. Additionally, neutrophil depletion was shown to attenuate acute rejection by reducing the recruitment of alloreactive memory CD8^+^ T-cells, thereby allowing Tregs to promote long-term graft survival in a mouse skin transplant model [72]. It has been demonstrated that neutrophils expressing Fas ligand and perforin can stimulate the production of CCL1, CCL2, and CCL5, considered to be T-cell chemoattractants, which leads to the recruitment of CD8^+^ T-cells [73]. In AMR, neutrophils are likely recruited to grafts by complement-fixing antibodies or by platelets adhering to vascular endothelium damaged by alloantibodies [74].

**Figure 3:**
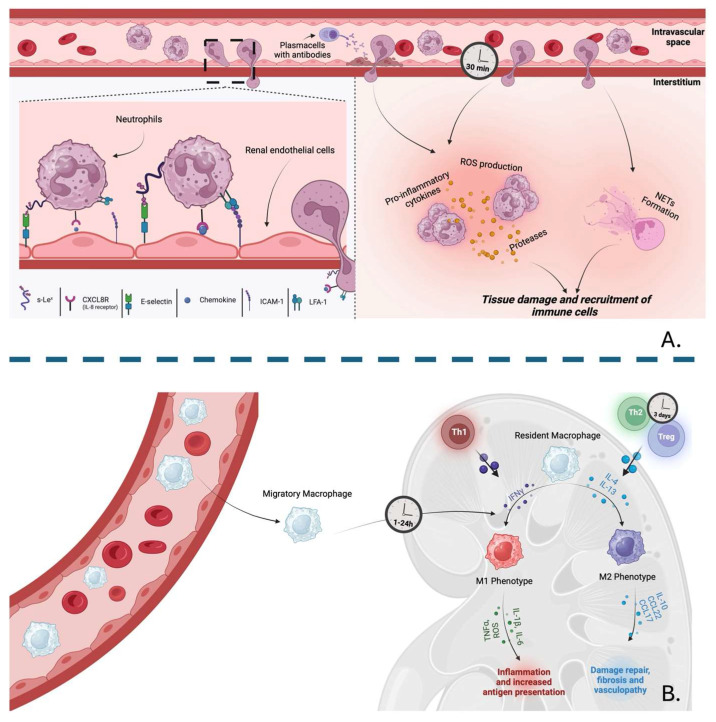
Macrophages and neutrophils infiltrate the transplanted kidney following ischemia/reperfusion injury. During ischemia/reperfusion injury, renal endothelial cells upregulate adhesion molecules, promoting increased neutrophil adhesion and recruitment. Activated neutrophils release cytokines, proteases, ROS, and extracellular traps, amplifying inflammation and tissue injury (**A**). Macrophages polarize into pro-inflammatory M1 or profibrotic M2 phenotypes, contributing, respectively, to acute rejection and chronic allograft dysfunction (**B**). Created with BioRender.com. Troise, D. (2025) https://BioRender.com/hz50wol [accessed on 1 September 2025].

In transplanted patients, an increased spontaneous NETosis occurs compared to donors, regardless of whether they experienced rejection or not. In the context of acute AMR, the Fc antibody region can bind to receptors on neutrophils, further increasing NETs production [75]. Additionally, neutrophils can also provide helper signals crucial for B-cell stimulation in the marginal zone of the spleen by secreting high levels of BAFFs and facilitating antibody class-switching, plasma cell differentiation, and survival [76]. Using animal models of kidney transplantation, the role of neutrophils in AMR has been linked to their depletion and reduced inflammatory cell infiltration, C4d, and IgG deposition. Furthermore, neutrophil depletion significantly decreased DSA levels after transplantation as well as BAFF and APRIL expression, suggesting novel insights into AMR-induced graft damage and its potential clinical management [77]. In chronic rejection, the accumulation of Th17 cells results in IL-17 production and subsequent neutrophil infiltration. Mutation in the IL-17 receptor is functionally associated with airway neutrophilia, which contributes to chronic rejection in lung transplant patients [78]. Recently trogocytosis, a biological process that involves the acquirement of a membrane fragment from a donor cell triggered by antigen receptor signaling, has been linked to graft damage, further strengthening the suggestion that the allograft rejection involves neutrophils through contact-dependent trogocytosis [79] (Figure 3).

### 3.3. Natural Killer Cells

NK cells can be classified into two subsets based on CD56 expression levels: low-density subset (CD56^dim^) and high-density subset (CD56^bright^), which differ in their distribution and functions. CD56^dim^ cells are the predominant subset in peripheral blood and exhibit strong cytotoxic activity. In contrast, CD56^bright^ cells are mainly found in secondary lymphoid organs and peripheral tissues, where they contribute to the immune responses by secreting pro-inflammatory cytokines, such as TNF and INF-γ [80].

The role of NK cells in kidney IRI models has been linked to the promotion of tubular injury, independent of INF production, due to their ability to directly kill tubular epithelial cells (TECs). This is facilitated by costimulatory molecules like retinoic acid early inducible-1 (RAE-1) on TECs, which activate the NKG2D ligand on NK cells. IRI upregulates both RAE-1 and NKG2D, enhancing NK responses and leading to perforin-dependent TEC killing. Also, NK cells can directly engage via activating molecules expressed on the damaged epithelium, such as CD137L on TECs with CD137^+^ NK cells, resulting in the induction of CXCL2 expression in TECs. This process leads to neutrophil recruitment and immune-mediated progression of tubular damage [81].

NK cell infiltration around blood vessels and the interstitium of a transplanted kidney increases following prolonged cold ischemia [82]. Their depletion has been associated with reduced neutrophil infiltration and lower production of pro-inflammatory mediators [83]. NK cells can contribute to the pathogenesis of both acute and chronic TCMR and AMR through various mechanisms [84]. For example, DSA-negative biopsies from patients with acute TCMR have shown an increased number of CD56^bright^ and CD57^+^ cells in the interstitial compartment and therefore suggested that interstitial inflammation and tubulitis are key features of TCMR. Conversely, in DSA-positive biopsies, increased levels of CD56^dim^ cells and antibody cellular-dependent cytotoxicity in the glomerular compartment were observed, suggesting that different patterns of NK cell marker expression may be associated with the involvement of distinct immune system pathways in graft rejection [85]. Interestingly, using extracted renal lymphocytes from human kidney transplant biopsies, increased numbers of CD56^bright^ NK cells were observed, while AMR patients had increased levels of both CD56^bright^ and CD56^dim^ NK cells, which were associated with enhanced levels of perforin, granzyme A, and granulysin in supernatants of AMR-derived biopsies, further supporting the suggestion about the specialized functional role of NK cells in different types of allograft rejection [86].

Microarray transcriptomic data were utilized to identify genes expressed in AMR within kidney graft biopsies. The analysis revealed significant enrichment of NK cell pathways and increased NK cell infiltration in AMR compared to TCMR and normal biopsies. Additionally, there was an increase in other leucocyte types, such as CD4^+^, CD8^+^ T-cells and DCs, but this data does not differentiate between AMR and TCMR, suggesting that NK cells have a pivotal role in AMR and graft failure after kidney transplantation [87]. Furthermore, transcriptome profiles of human allograft biopsies from chronic active AMR, active AMR, and TCMR revealed that chronic active AMR is characterized by an enrichment of genes related to NK cell-mediated cytotoxicity, with a higher proportion of CD56^bright^ NK cells compared to active AMR biopsies. NK cells and T-cells were the most abundant infiltrating cells in chronic active AMR and TCMR, while neutrophils and monocytes were most prevalent in active AMR [88].

### 3.4. The Complement System

IRI-induced DGF is regulated by the complement system. Transcriptomic analysis of kidney allograft biopsies revealed activation of the complement cascade in deceased donors prior to organ retrieval and before the cessation of blood circulation [89].

Several studies on complement activation in deceased donors have focused on complement fraction C3 [90,91]. Moreover, C3, the most abundant component of the complement system, is considered the convergence point of all three major pathways: the classical (CP), alternative (AP), and lectin (LP) pathways [92]. However, recent evidence showed that both plasma levels of C5a and C5b-9 (also known as membrane attack complex, MAC) are also significantly elevated, suggesting that downstream complement products are equally important. Additionally, C5b-9 has been associated with an increased risk of rejection after transplantation [93,94], and perioperative measurements could be used as a clinical biomarker of DGF [95]. Additionally, the pericyte-to-myofibroblast transition has been associated with complement activation during renal IRI. In particular, C5a has been demonstrated to have a profibrotic effect by influencing the TGFβ pathway, resulting in extracellular matrix deposition and the transition of tubular cells to a profibrotic phenotype [96].

Different organs may activate the complement system through different mechanisms. LP is primarily involved in complement activation in the ischemic heart and intestine, whereas the AP is considered the most common activation pathway in the kidney [97], as demonstrated by the finding that the inhibition of factor B (FB), an essential component of the alternative pathway, led to reduced kidney reperfusion damage after warm ischemia in mice [98].

Once again, DAMPs released due to IRI may lead to the activation of the complement cascade and the final formation of the MAC, resulting in direct injury to the kidney by inducing apoptosis in TECs. Notably, underlying nephropathies (such as atypical hemolytic uremic syndrome, C3 glomerulopathy, and diabetes) and maintenance hemodialysis have been reported to cause complement activation in recipients even before transplantation [99]. However, the AP may also play a protective role in renal IRI, as evidenced by the detrimental effects on the kidney in the absence of properdin, a positive regulator of the AP. Properdin, produced by TECs, is crucial for the opsonization of damaged cells, the proper clearing of apoptotic cells, and the reduction in inflammation [100]. Although the AP is considered the most frequently activated pathway during renal IRI, some research has shown that TECs overexpress collectin-11 (CL11), a soluble pattern recognition receptor that can activate mannan-binding serine protease-2 (MASP-2) after binding to fucosylated molecules exposed on the surface of tubular cells following ischemic injury, leading to the initiation of the LP [101,102]. Findings demonstrated that administration of a bolus of L-fucose can prevent CL11 binding to TECs, due to the obstruction of the carbohydrate recognition site on CL11, thereby reducing complement activation and damage in an IRI animal model [103]. This suggests that the LP might also play a role in the aberrant complement activation during renal IRI.

Additionally, the massive release of C3a and C5a (anaphylatoxins) derived from the cleavage of C3 and C5 promotes the release of pro-inflammatory cytokines, ROS, and chemokines, as well as inflammatory cell recruitment after binding their receptors, C3aR and C5aR, respectively. Furthermore, complement system activation increases APCs’ priming activity on T-cells by increasing the expression of costimulatory molecules and antigen presentation [104]. Interestingly, DCs are able to synthesize C3, which is required for the stimulation of T-cell responses in vivo and in vitro [105]. Additionally, TLR engagement after IRI induces DCs’ phenotypic and functional changes, converting them from antigen-scavenging cells to mature DCs capable of presenting antigen to T- and B-cells. These characteristics highlight the role of DCs and the complement system as a bridge between innate and adaptive immune responses [96]. In C3, FB, or C3aR knockout mice, DCs have been shown to be incapable of upregulating MHC class II, leading to decreased T-cell priming and a diminished alloreactive T-cell response [106]. Contrarily, downregulation of the complement regulatory protein decay-accelerating factor in DCs leads to increased expression of C3a and C5a, thereby enhancing T-cell proliferation [107]. Also, some complement components like C1q, C3, and C4 have been shown to exert immunomodulatory effects on APCs, promoting the development of tolerogenic DCs [108]. Furthermore, T-cells also express intra- and extracellular anaphylatoxin receptors, and their engagement is associated with inflammatory responses and production of IL-12, necessary for Th1 activation [109].

Complement activity is considered a crucial factor in the development of TCMR, and its role is not limited to the extracellular space. Recent studies have shown that intracellular complement activity regulates basic cellular metabolic processes, thereby maintaining T-cell homeostasis. Studies reported that the protease cathepsin L (CTSL) in T-cells continuously cleaves C3 to generate C3a, which interacts with C3aR via the mTOR pathway, required for cell survival and the induction of effector T-cell responses. Moreover, the use of a CTSL inhibitor resulted in decreased C3 activation, inducing T-cell apoptosis within 8–12 h [110]. In human T-cells, the activation of the CD46 intracellular domain CYT-1, a complement regulator that binds and inactivates C3b and C4b, is associated with the enhanced expression of GLUT1, increased glycolysis, and the activation of the checkpoint kinase mTORC1, inducing Th cell responses [111]. Intracellular C5 activation is considered another important regulator of CD4^+^ T-cell responses. The C5a-C5aR1 signaling pathway acts as a positive regulator for the induction of IFN-γ production and Th1 differentiation, while C5aR2 is considered a negative regulator of this process [112].

Complement-dependent cytotoxicity and the deposition of C4d in peritubular capillaries of renal allografts suggest the involvement of the complement in the development of AMR [113,114]. The incompatibility of AB0 blood group antigens and the human leukocyte antigen (HLA) system is involved in the development of acute and chronic AMR through DSAs. In addition, the development of de novo DSAs could occur in a T-cell-dependent manner in transplant recipients [115]. These antibodies are considered strong activators of complement via CP through C1q engagement, leading to endothelial damage [116]. MAC formation has been shown to upregulate inflammatory genes in ECs. Furthermore, the insertion of the MAC can enhance the capacity of ECs to recruit CD4^+^ T-cells and increase INFγ production [117]. Furthermore, the assembly of MAC also induces a prothrombotic state through the production of tissue factor, von Willebrand factor, and the upregulation of P-selectin on the surface of ECs [118]. Linear C4d deposition in peritubular capillaries or medullary vasa recta, long considered a marker of AMR, is no longer a prerequisite for suspecting AMR according to the current Banff criteria. An update of the Banff 2022 states that, in the presence of circulating DSAs, lesions of microvascular inflammation without C4d deposition in peritubular capillaries probably indicate antibody activity and thus the development of AMR [119].

**Figure 4:**
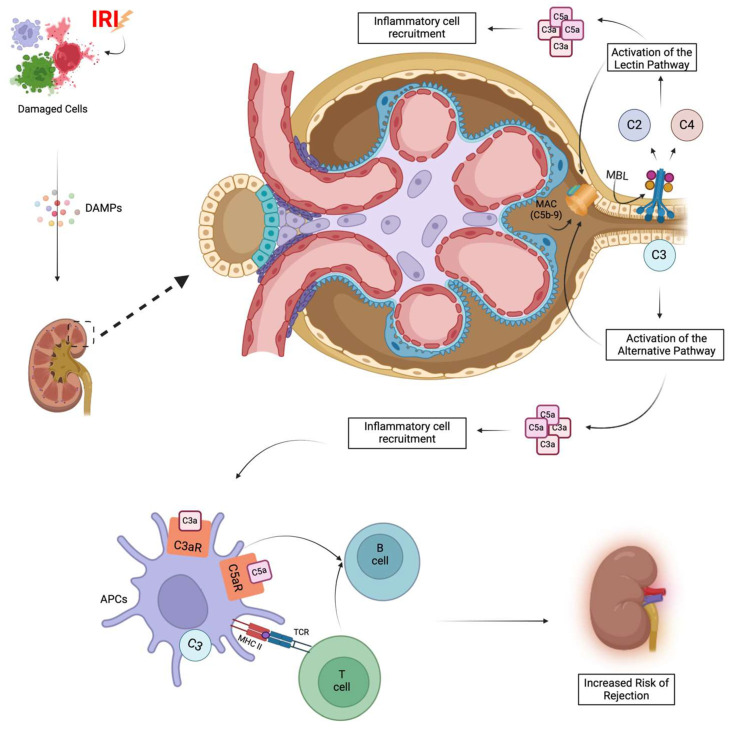
Complement system activation has a role in the development of allograft rejection. Renal ischemia/reperfusion injury activates the complement system, primarily through the alternative pathway, leading to MAC formation and direct cellular injury. Complement activation releases C3a and C5a, driving cytokine and chemokine production, inflammatory cell recruitment, and antigen-presenting cell activation, thereby enhancing T- and B-cell responses and increasing the risk of rejection. Created with BioRender.com. Troise, D. (2025) https://BioRender.com/hvu1927 [accessed on 1 September 2025].

In summary, complement system activation plays a role in the development of DGF in the context of IRI and the increased risk of allograft rejection. Moreover, recent studies demonstrated that complement has a crucial role in supporting B-cell viability [120]. Therefore, a better understanding of the activation and functions of the complement system might be helpful to expand the blueprint for kidney transplantation therapy in the context of IRI and rejection (Figure 4).

## 4. Dendritic Cells

DCs play a pivotal role in coordinating human immunity, providing a vital connection between innate and adaptive immune responses. The DCs/T-cell interaction can lead to opposite outcomes: activation or inhibition of the immune responses, resulting in increased immunogenicity or tolerance. The outcomes appear to be determined by the origin of DCs and their activation state, as activated mature DCs induce T-cell immunity, while resting non-activated DCs can induce tolerance [33].

During IRI, DCs, neutrophils, and NK cells interact to amplify tissue damage through the secretion of chemokines and cytokines and direct cell-to-cell contact. In the context of renal IRI, DCs become activated within the kidney parenchyma and become the primary producers of TNFα, promoting kidney TEC apoptosis, endothelial damage, and fibrosis. Upon reperfusion, intrarenal DCs upregulate maturation markers, including major histocompatibility complex (MHC), CD80, CD86, CD40, and CD1d, which makes them able to present self-antigens to T-cells [121]. DCs migrate from the injured kidney to the draining lymph nodes within 24 to 48 h from IRI and present antigens to T-cells, particularly naïve antigen-specific CD8^+^ cells, facilitating the effective induction and activation of adaptive immunity [122]. Furthermore, evidence suggests that in renal IRI, pro-inflammatory cytokines and TNF produced by hypoxic ECs can recruit DCs and that HIF-1α promotes their maturation, leading to impaired kidney function [123]. In fact, hypoxia during IRI may affect DC quality and the intensity of immune response by the upregulation of HIFs.

In kidney grafts, donor DCs have been shown to directly induce acute allograft rejection by the direct allorecognition pathway. In contrast, recipient DCs, with their longer lifespan, can drive chronic kidney rejection through semi-direct and indirect pathways [124,125]. Specifically, the adaptive immune responses are initiated by DCs through three different pathways: direct, semi-direct, and indirect allorecognition. In the direct allorecognition pathway, donor DCs directly present the donor MHC–antigen complex to recipient T-cells; the indirect pathway is characterized by recipient DCs capturing donor antigens and presenting them to recipient T-cells. In addition, the semi-direct pathway, also known as “cross-dressing”, involves recipient DCs acquiring the intact donor MHC I-antigen complex and presenting it to recipient CD8^+^ T-cells in secondary lymphoid organs [126].

The activation of the T-cell receptor (TCR) by the MHC–antigen complex on DCs, the binding of T-cell adhesion molecules to ICAM and VCAM on DCs, and co-stimulatory signals (the interaction between CD28 on T-cells and B7 molecules on DCs) are considered the three key mechanisms by which DCs and T-cells interact with each other. In addition, the expression of both MHC class I and class II molecules on DCs makes them capable of activating both CD4^+^ and CD8^+^ T-cells [127]. RNA sequencing on kidney allograft biopsies with TCMR has shown increased levels of DCs in patients with rejection compared to those without rejection. Interestingly, the median time from transplantation to TCMR in the study was 12 months, raising questions about the origin of intragraft DCs during TCMR, since donor DCs are typically replaced by recipient DCs within 24 h to 7 days after surgery [128,129]. Another aspect to consider is that recipient DCs infiltrating the transplanted organ preferentially promote T-cell differentiation into a Th1 phenotype, which has a pivotal role in rejection, and also that antigen presentation by recipient DCs is not restricted to the indirect allorecognition pathway, as they are also effective at priming directly alloreactive T-cells [130]. In a recent cross-sectional study analyzing data from AMR, TCMR, and mixed (TCMR and AMR together) rejection, nine types of intragraft immune cells were assessed. Among these, DCs showed an increased proportion among rejection phenotypes compared to the no rejection group [131].

Immature DCs can be activated by antigens released as a result of IRI and function as APCs, triggering B-cell activation through helper T-cells [124]. Specifically, after recipient DCs capture donor antigens within the transplanted organ, they migrate to the secondary lymphoid organs. There, these DCs, or donor antigens transferred via exosomes, trogocytosis, or similar mechanisms, present the processed antigens to alloreactive CD4^+^ T-cells. Subsequently, some of them differentiate into T follicular helper (Tfh) cells and migrate to the T–B-cell interface, leading to B-cell entry into germinal centers, where they undergo somatic hypermutation and class-switch recombination, resulting in the production of memory B-cells and plasma cells and contributing to the development of antibody-mediated allograft rejection [130].

**Figure 5:**
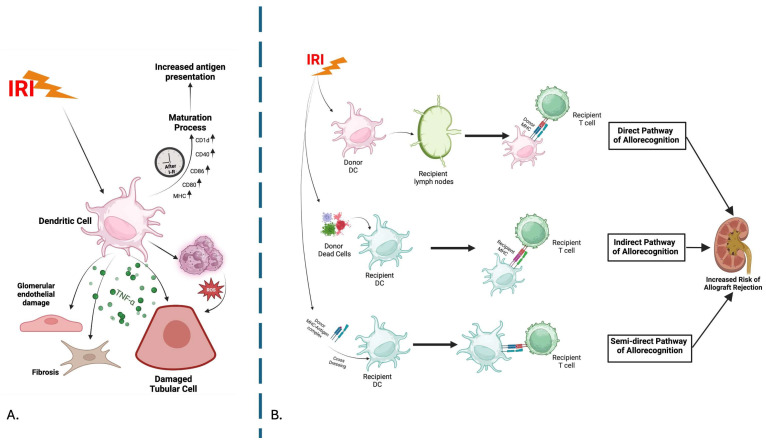
Dendritic cells coordinate the immune responses and mediate the damage in ischemia/reperfusion injury. During ischemia/reperfusion injury, dendritic cells (DCs) mature and produce TNFα, contributing to endothelial and tubular injury, fibrosis, and neutrophil-derived ROS, while enhancing antigen presentation (**A**). In addition, the three distinct pathways (direct, indirect, and semi-direct) can initiate the adaptive immune responses, potentially increasing the risk of allograft rejection (**B**). Created with BioRender.com. Troise, D. (2025) https://BioRender.com/gkesib6 [accessed on 1 September 2025].

Therefore, DCs have a pivotal role in the interaction between IRI and rejection. Understanding the mechanisms for DC involvement holds great promise for developing new therapeutic strategies to enhance transplant outcomes (Figure 5).

## 5. Adaptive Immune Responses Involved in the Development of IRI and Rejection

### 5.1. T-Cells

T-cells, in addition to innate immune responses, have recently been considered to have roles in both perpetuation of damage and its repair, potentially influencing the transition from ischemic AKI to chronic allograft injury. Studies have investigated the role of T-cells in the pathogenesis of IRI, demonstrating that CD4^+^/CD8^+^ T-cell knockout mice were protected from IRI 48 h post-ischemia and that this protection was associated with both reduced neutrophil infiltration and decreased tubular necrosis [132]. Moreover, T-cell infiltration is considered an early event after IRI, as shown by other researchers, who demonstrated kidney infiltration of CD4^+^ T-cells just one hour after reperfusion [133]. Most T-cells can be distinguished by their unique transcription factor signatures, cytokine production profiles, and effector functions.

Th1 responses are coordinated by t-bet, a member of the t-box transcription factor family, which regulates the differentiation and functions of immune cells. After IRI, TECs release IL-18, which promotes the conversion of CD4^+^ naïve T-cells into Th1 cells. This process is associated with INFγ and TNFα secretions and the recruitment of other immune cells, including NK cells and neutrophils [134,135,136,137]. Additionally, DGF is characterized by an increase in renal Th1 cells and the cytokines they release, which promote the activation of macrophages, the production of lysosomal enzymes, and ROS [138]. Th1 and Th2 cells likely exert opposite effects; studies in IRI murine models suggested that Th1 cells are pathogenic, whereas Th2 cells are protective [139]. Furthermore, Th1 cells, through the secretion of pro-inflammatory IL-2, IL-12, INF-γ, and TNF-α, promote alloimmune responses in the graft, increasing its immunogenicity. Specifically, INF-γ has been shown to promote antigen processing and presentation, stimulate macrophage and NK cell activity, and increase T-cell allograft infiltration, thereby promoting acute rejection processes [140,141]. Equally important is the observation that both antibody-mediated and T-cell-mediated acute rejections are linked to elevated levels of donor-reactive IFN-γ and IL-21 memory T-cells before and 3–6 months after kidney transplantation [142]. These findings were supported by a meta-analysis, which concluded that patients with a high frequency of donor-reactive IFN-γ ELISPOT are at increased risk for acute kidney rejection [143]. Conversely, Th2 cells have traditionally been associated with the production of anti-inflammatory cytokines, such as IL-4 and IL-13, both capable of antagonizing pro-inflammatory INF-γ and TNF-α while promoting antioxidant and antiapoptotic functions [144,145]. Specifically, Th2 responses are orchestrated by the transcription factor GATA-binding protein 3 (GATA3) and are characterized by the release of cytokines, including IL-4, IL-5, IL-10, and IL-13, contributing to protective effects after IRI [146]. Notably, IL-4 treatment or secretion has been shown to enhance allograft survival due to the promotion of Th2 responses, a reduction in Th1 frequency, and the expansion of the Treg compartment [147].

Additionally, IL-17 produced by Th17 cells under the control of transcription factors STAT3 and retinoic acid receptor-related orphan receptor-γt (RORγt) has been shown to recruit other immune cells like neutrophils, exacerbate inflammation and tissue fibrosis, and contribute to the progression of chronic kidney disease. Increased levels of Th17 were identified between 3 and 7 days after IRI. The recruitment and activation of Th17 cells in the kidney is facilitated by cytokines produced by TECs and DCs, including IL-1, IL-6, and IL-23, causing lymphocyte shift towards Th1 phenotypes [146]. Interestingly, elevated IL-17 levels have also been linked to allograft rejection. Notably, increased infiltration of Th17 cells is significantly associated with the severity of TCMR, decreased allograft function, and severe kidney interstitial and tubular injury. The production of inflammatory mediators by TECs exposed to IL-17, increased neutrophil recruitment, and the enhancement of early alloimmune responses are considered some of the proposed mechanisms to explain these findings during IRI and allograft rejection processes [148]. The extent of Th17 cell infiltration in kidney biopsies from patients with TCMR has been assessed, showing that Th17 graft infiltration could serve as an indicator of transplantation prognosis and response to anti-rejection therapy [149]. Furthermore, some studies have demonstrated that patients with reduced kidney function post-transplantation exhibit increased Th17 infiltration compared to Tregs and a lower Treg/Th17 ratio, suggesting that the imbalance in T-cell subtype is linked to chronic kidney disease in kidney transplanted patients [150].

During the recovery phase, CD4^+^ Tregs may reduce inflammation through the release of IL-10 and TGFβ, thereby reducing the production of IL-1, TNFα, and IFNγ, and decreasing overall CD4^+^ T-cell proliferation. Both peripheral and resident Tregs have been demonstrated to play a role in attenuating injury after IRI [151,152,153]. The transcription factor FOXP3 serves as a lineage specification factor of Treg cells. Tregs express higher levels of CD5, CTLA4, and IL-2 receptor α-chain (CD25) and exhibit potent suppressor activity [154]. Another study aimed to investigate the role of programmed death ligands (PD-L1 and PD-L2); it was observed that blocking PD-L1 or PD-L2 led to increased kidney damage and inflammation due to the decreased protective ability of Tregs in IRI [155].

Th1, Th2, Th17, and Treg subsets belong to αβ T-cells and constitute the majority of peripheral T-cells, while γδ T-cells, found in skin and the gut epithelia, constitute a smaller fraction. It has been demonstrated that γδ T-cells might play a role in the migration of αβ T-cells in a kidney IRI murine model, but their depletion does not prevent injury [156]. Conversely, other studies pointed out that the number of γδ T-cells in the kidney was negatively correlated with the estimated glomerular filtration rate, suggesting that γδ T-cells may exacerbate ischemic AKI. Ultimately, even though the role of γδ T-cells in IRI remains unclear, they likely have less impact on the pathophysiology of IRI compared to the numerous αβ T-cells [157].

The role of CD8^+^ T-cells in ischemic injury is less well defined. Initially, no significant pathogenic role was reported; later, CD8^+^ T-cell depletion was found to be associated with increased intrarenal TNFα production and a reduction in anti-inflammatory macrophages. However, the role of CD8^+^ T-cells still needs to be elucidated [158].

The extent of CD8^+^ T-cell, or cytotoxic T-cell (CTL), infiltration has been found to be correlated both with allograft survival and allograft damage after direct activation via MHC class I antigen presentation. CTLs can infiltrate the allograft and secrete pro-inflammatory TNF-α and IFN-γ, induce apoptosis via perforin and granzyme production, and activate NK cells, leading to allograft injury. Risk of acute rejection has been correlated with increased frequency of effector memory CTLs. Established hypotheses suggest that CD8^+^ T-cells can enhance inflammatory responses by increasing Th1 and suppressing Th2 cytokine production [159].

**Figure 6:**
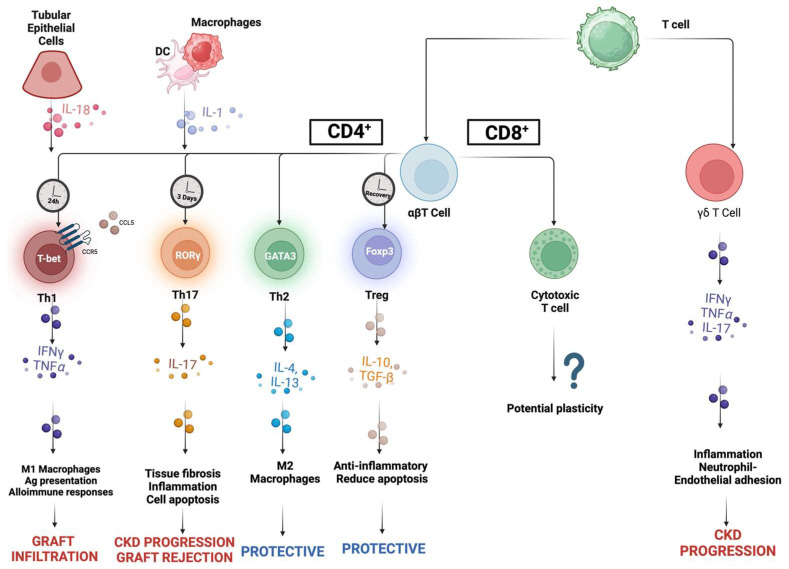
The role of T-cells in ischemia/reperfusion injury. T-cells infiltrate the kidney early after ischemia/reperfusion injury and play a central role in rejection. While most belong to the αβ T-cell subsets (Th1, Th2, Th17, Tregs), smaller γδ T-cells are primarily found in skin and gut epithelia. Th1 cells promote graft immunogenicity, whereas Th2 cells appear to have protective anti-inflammatory effects. The role of CD8^+^ T-cells remain less defined but may involve functional plasticity during injury and rejection (uncertain, hence question mark). Created with BioRender.com. Troise, D. (2025) https://BioRender.com/6yhjdan [accessed on 1 September 2025].

Other researchers examined a cohort of kidney transplant patients with allograft rejection, discovering that AMR was associated with a significant increase in the number of CD28^−^CD8^+^ T-cells in the peripheral blood at the time of biopsy, suggesting that CD28^−^CD8^+^ T-cells may play a role in the pathobiology of AMR [160]. Conversely, a recent study revealed a temporal shift in the gene expression of kidney graft-infiltrating CD8^+^ T-cells from a cytotoxic to a regulatory-like phenotype within 3 weeks after kidney transplantation in mice that spontaneously accepted kidney allografts, suggesting potential plasticity in these cells. Further investigations are needed to explore the role of CD8^+^ T-cells in allograft rejection mechanisms [161] (Figure 6).

### 5.2. B-Cells

While the effect of T-cells could be either pathogenic or protective depending on the specific subsets involved, B-cells have been found to be predominantly pathogenic. After B-cells mature in the bone marrow, they migrate to lymphoid organs and become activated in the spleen or other secondary lymphoid organs after the recognition of an antigen through the B-cell receptor (BCR). Specifically, after the binding of alloantigens by BCR and the engagement of co-receptors, such as complement or TLRs, the antigens are internalized, processed, and presented on MHC class II molecules to CD4^+^ T-cells. B-cells then migrate to the T–B-cell interface and establish interactions with follicular helper T (Tfh) cells that have been previously activated by DCs, resulting in the extrafollicular differentiation of B-cells into memory B-cells, short-lived plasma cells, or entry into a germinal center response for class-switching and somatic hypermutation to select high-affinity B-cells [162,163].

During IRI, activated B-cells accumulate in the renal tissue due to chemokine effects and start to produce antibodies, which may bind allograft antigens, exacerbating IRI [164,165]. Furthermore, B-cells can be classified as B1 cells and B2 cells [166]. B1 cells account for about 5–10% of the total B-cell population and are located in the pleural and peritoneal cavities, where they secrete polyreactive IgM antibodies in response to T-cell-dependent antigens. During IRI, damaged TECs release DAMPs that activate B1 cells as part of the innate immune response, leading to increased damage due to the release of antibodies and cytokines [167]. Conversely, B2 cells accumulate in the kidney due to chemokines like CXCL13 released by damaged TECs, contributing to the inflammatory and immune responses through the release of more cytokines and chemokines [168]. Leukocyte responses in a mouse model of renal IRI have been investigated, finding elevated levels of circulating and renal-infiltrating B-cells that produce the chemokine CCL7, promoting inflammatory cell recruitment to the injured kidney. Moreover, blocking CCL7 in mice led to reduced myeloid cell infiltration into the kidney and improved IRI-related AKI [169]. Similar findings have been obtained in other studies using murine lung hilar clamping; the production of monocyte chemokine CCL7 by lung-infiltrating B-cells has a pivotal role in monocyte recruitment and neutrophil extravasation, which contribute to deteriorating lung function. In addition, the authors showed that the combined activation of the B-cell receptor (BCR) and TLR4 on B-cells is essential for monocyte recruitment to the damaged lung, suggesting that B-cells are critical in the progression of lung IRI [165].

Initially, B-cells have not been considered one of the main players in the induction of late rejection or tolerance in organ transplantation, largely due to the prominent role of cellular immunity in the development of early graft rejection, even though their role in the pathogenesis of allograft damage was recognized from the beginning [170,171]. Today, post-transplant and de novo DSAs represent a key risk factor for long-term allograft loss due to their major role in both acute and chronic AMR through antibody production against HLAs and non-HLAs. However, certain B-cell subtypes are also known for their regulatory function, producing IL-10, IL-35, or TGF-ß. Also, B-cells are now recognized for their role not only in antibody production and their response to tissue injury, as observed in IRI, but also for their function as antigen-presenting cells [172]. Research has demonstrated in a murine model of vascularized heart allografts that signs of chronic allograft vasculopathy were present in mice lacking antibodies but not in those without B-cells, suggesting that chronic rejection can occur in the complete absence of antibodies. Moreover, they suggested that B-cells contribute to this process by maintaining splenic lymphoid architecture, promoting T-cell cytokine production, and facilitating infiltration of T-cells into graft vessels [173]. In contrast to other APCs, including DCs, B-cells can only present antigens through the indirect allorecognition pathway. After alloantigen engagement, B-cells internalize and process antigens for presentation on MHC class II molecules, then interact only with CD4^+^ T-cells because they cannot stimulate direct CD8^+^ T-cell responses due to the lack of the cross-dressing allorecognition pathway [174]. Notably, Sarwal et al. [175] and later Liarski et al. [176] suggested that infiltrating B-cells contribute to TCMR by acting as APCs for T-cells within the allograft. The latter, in particular, developed computational tools to measure the spatial relationships between different cell subtypes in tissue. They used these tools to identify B-cell-Tfh cell cognate interactions in human renal biopsies from patients with TCMR and those exhibiting cellular rejection and C4d deposition, observing that the majority of Tfh cells were interacting with B-cells in biopsies with cellular rejection and C4d deposition. In addition, they showed that this cell interaction in patients undergoing rejection was associated with functional Tfh cells expressing higher Bcl-6 and IL-21 levels, considered regulators of Tfh differentiation and function [176]. Another study has shown that intrarenal B-cells can stimulate naïve T-cells but have a reduced capacity to promote the expansion of Tfh cells, suggesting that intrarenal B-cells during transplant rejection are transcriptionally distinct from those in lymph nodes [177].

AMR is believed to cause ~50% of kidney graft losses each year [178]. In kidney transplantation, this condition is characterized by the presence of circulating DSAs, histological signs of antibody interactions with the vascular endothelium, such as C4d deposition, and microvascular inflammation and injury. Moreover, chronic AMR is histologically identified by the presence of glomerulopathy, multilamination of the peritubular capillary basement membrane, and/or arteriopathy characterized by intimal fibrosis. In this context, IRI can be a critical risk factor, promoting oxidative stress and tissue damage that activate B-cells, increasing their production of DSAs, and leading to further immune attacks on the kidney [179] (Table 1).

## 6. Prevention of IRI and Treatment Strategies for Immunological Consequences

Therapeutic strategies against IRI are warranted, as they can play a crucial role in mitigating T-cell and antibody-mediated kidney rejection. The demand for kidney grafts currently far exceeds the available supply, making it essential to develop innovative and novel therapeutic strategies to expand the applications of the deceased donor pool. Therefore, kidney grafts from extended criteria donors (ECDs) and those donated after cardiocirculatory death (DCD) are viable options to address the increasing demand for transplants. However, grafts from deceased donors, particularly ECDs, are more susceptible to IRI and therefore to DGF and kidney allograft rejection. This intrinsic vulnerability directly links IRI to the critical unmet need for donor organs [180].

Organ procurement, preservation, and transplantation lead to a series of physiopathological stresses on allografts. A systematic review and meta-analysis on graft preservation techniques suggested that machine perfusion and normothermic regional perfusion (NRP) are effective techniques to mitigate IRI. Particularly, machine perfusion was associated with a reduced incidence of DGF, acute rejection, and graft failure [180].

Additionally, NRP, which temporarily restores blood flow after the declaration of death via arterial and venous cannulae inserted either into the femoral vessels or directly into the aorta and vena cava following a rapid laparotomy, is considered a safe alternative to in situ cooling and rapid procurement for kidneys from DCD donors, as recommended by a consensus statement from several government agencies [181,182]. Notably, Kerforne et al. demonstrated that kidneys transplanted after 4 to 6 h of NRP exhibited improved function and outcomes compared to those without NRP. Nevertheless, the optimal NRP protocol has yet to be fully defined, and this requires further investigations [183].

Another strategy that has emerged to help prevent AKI during the transplantation process is ischemic preconditioning (IPC), which involves cycles of ischemia and reperfusion before a potentially sustained and more pronounced ischemic injury occurs. IPC was first introduced in 1986 using an animal model, conducting a series of 5 min circumflex artery occlusions, each followed by 5 min of reperfusion, before a sustained 40 min occlusion, with the following presentation of a 75% reduction in myocardial infarction compared to the control group [184]. Thereafter, IPC has been shown to protect cells from injury by reducing ROS production, improving renal blood flow, and inducing antiapoptotic proteins (Bcl-2 and Bcl-x) via the Akt signaling pathway. Additionally, IPC has been involved in the reduction in renal inflammation by decreasing the production of pro-inflammatory cytokines, such as TNF-α, IL-1β, IL-6, MCP-1, and IL-17 [185].

It has been demonstrated that an increase in blood levels of inflammatory neutrophils and monocytes occurs in mice subjected to focal cerebral ischemia. It has also been found that IPC prevents the rise in pro-inflammatory cytokines, increases the anti-inflammatory/pro-inflammatory ratio, and reduces brain infarct volume and ischemia edema, leading to the coordination of immune responses and host defense [186]. However, a meta-analysis investigated the protective role of IPC in kidney transplantation among 1145 kidney transplant patients, finding that the estimated glomerular filtration rate was improved in the IPC group three months after transplantation but not after one year, suggesting that IPC does not have significant effects on lowering serum creatinine. Notably, they found no significant differences between the groups in terms of acute rejection rates and graft loss [187]. Summarizing the findings above, implications exist that IPC has the potential to protect the kidney from IRI and reduce the risk of DGF and graft loss due to rejection processes. However, some limitations need to be further addressed due to inconsistent results, indicating the need for additional data to improve our knowledge gaps.

Considering the role of Treg cells in IRI, adoptive cellular transfer of Tregs can be a promising therapeutic approach to alleviate IRI-induced inflammatory responses in kidney allografts. The strategy is linked to promising results from Treg cellular therapy in treating autoimmune diseases and promoting immune tolerance in organ transplantation [188]. The infusion of polyclonally expanded autologous Tregs after IRI has been shown to increase Treg trafficking to the renal interstitium, thereby reducing the infiltration of innate and adaptive immune cells, such as DCs and T-cells. Consequently, the release of anti-inflammatory cytokines by activated Tregs suppressed IRI-induced inflammatory responses, leading to reduced kidney damage and promoting recovery [189].

In kidney transplantation, immunosuppressive regimens are generally categorized into induction and maintenance therapies. The two main induction drugs are basiliximab and anti-thymocyte globulin (ATG). Basiliximab is a chimeric mouse-human monoclonal antibody that targets the α chain (CD25) of the IL-2 receptor on T-cells, while ATG is a polyclonal antibody that primarily targets T-cells and is known to improve outcomes for patients with high immunological risk due to its ability to deplete lymphocytes [190]. The current standard for maintenance immunosuppression includes calcineurin inhibitors (CNIs), mTOR inhibitors (mTORis), and anti-proliferative drugs like mycophenolate mofetil with or without corticosteroids [191]. Unfortunately, many of these treatments influence the number and function of Tregs. The viability and cytokine production of Treg cells in heart transplanted patients were examined, finding that the use of basiliximab is associated with decreased Treg counts and function due to negative effects of the immunosuppressive drug on Tregs’ proliferation, expression of Foxp3, and IL-10 secretion capacity [192]. Notably, CNIs have been shown to reduce Treg numbers in kidney transplanted patients, while mTORis, by blocking CD4^+^ T-cell progression through the cell cycle, may actually support Treg function [193]. The effects of Treg modulation and Treg-based therapies are not yet fully understood, so a deeper understanding of the effects of how immunosuppressive therapies impact Tregs’ function and viability is mandatory for the development and application of future therapeutic strategies.

Given the significant role of B-cells in IRI and allograft rejection, current B-cell-targeting therapeutic approaches, utilizing drugs that directly eliminate or modulate B-cells or inhibit molecules essential for B-cell survival, may be of value in managing IRI and reducing rejection rates [37]. B-cell-depleting therapies target clusters of differentiation, which are specific transmembrane proteins, causing cell removal through antibody-dependent cytotoxicity, phagocytosis, complement-dependent cytotoxicity, or direct cell death. Specifically, anti-CD20 monoclonal antibodies (mAbs), such as rituximab or ofatumumab, deplete cells from pre-B to memory-B stages but do not affect progenitors, plasma blasts, and plasma cells. Moreover, there are anti-CD19 mAbs, which primarily target B-cells and CD20-negative plasma blasts, and anti-CD38 mAbs, used in the treatment of monoclonal gammopathies to target plasma cells. In addition, several therapies target molecules critical to B-cell homeostasis and function, such as BAFFs, APRIL, and Bruton’s tyrosine kinase inhibitors, all of which are involved in B-cell survival, proliferation, and activation [194].

Notably, in a mouse model of cardiac IRI, the use of anti-CD20 mAb reduced left ventricular remodeling and improved cardiac function [195]. Furthermore, B-cell depletion has been shown to protect mice from IRI-related intestinal damage in a mesenteric IRI model [196]. In conclusion, although some authors have pointed out the importance of modulating B-cell responses to prevent ischemia/reperfusion damage, conclusive data is still lacking.

However, given the higher immunologic risk associated with highly sensitized kidney recipients or ABO-incompatible kidney transplants, rituximab is commonly used in clinical settings to prevent AMR [197].

Complement-modulating therapies have shown a beneficial role in the treatment of immunological complications related to kidney transplantation. Also, complement activation can occur in different renal compartments. During IRI and TCMR, complement is deposited within the tubulointerstitial compartment, while during AMR, complement is deposited at antibody binding sites in the vascular compartment. Therefore, therapeutic agents should be carefully selected and targeted to the appropriate compartment to ensure the effectiveness of the treatment [198].

Recently, other researchers demonstrated that the inhibition of the innate immune cascade with a central complement component C3 inhibitor at the time of transplantation prevents the DSA-mediated tissue injury in a nonhuman primate model and inhibits T and B-cell activation and proliferation, suggesting that the complement cascade inhibition may have immunomodulatory effects beyond its direct impact on antibody-mediated injury [199].

In clinical practice, several other strategies for complement blockade are currently used, with some being used off-label and mostly for AMR, including eculizumab (a humanized anti-C5 mAb) that inhibits the terminal complement pathway and C1 esterase inhibitors that block the classical pathway activated by DSAs. Furthermore, factor B and factor D inhibitors have gained a lot of attention for their ability to selectively target the alternative pathway [200]. However, the role of complement-modulating therapies in the context of IRI is still limited. The results from two randomized pilot trials indicated that the administration of eculizumab in the operating room prior to reperfusion in patients receiving deceased donor kidney transplant for DGF prevention showed a difference in DGF rates or graft survival [201]. Conversely, a randomized controlled trial with 57 pediatric kidney transplant recipients showed that the administration of eculizumab prior to transplantation resulted in immediate graft function in all patients in the eculizumab group with lower rates of arteriolar hyalinosis and chronic glomerulopathy on biopsy [202].

Given the role of the complement system in transplantation, studies involving several organs and experimental models have garnered significant attention for its pivotal role in mediating damage during IRI and rejection. However, the findings are still contradictory, and further studies are needed to shed light on the subject.

Xenotransplantation, the transplantation of organs and tissues from genetically engineered pigs, is emerging as a potential solution to the shortage of human donor organs. In contrast to conventional transplantation, xenotransplantation permits modification of the donor organ in order to protect it from the human immune response. Moreover, in addition to organ transplantation, xenotransplantation has the potential to be used for a wide range of applications, such as the treatment of diabetes with pig islets, corneas for blindness, dopamine-producing cells for Parkinson’s disease, red blood cells for transfusion, and gene-edited heart valves. Importantly, it would increase access to high-quality grafts and reduce the need for living donors, representing a revolutionary advancement in medicine [203]. Specifically, integrating the treatment strategies into xenotransplantation may hold promise for enhancing graft viability and mitigating immune rejection. Interestingly, the combined use of ATG and C3 complement inhibitor, along with low-dose intravenous immunoglobulin and plasmapheresis, has recently enabled a xenotransplant recipient to survive until postoperative day 85 [204]. It has been proposed that three key aspects of conventional allograft IRI, including complement activation, sterile inflammation, and endothelial activation, play a particularly important role in xenograft ischemia/reperfusion injury. As demonstrated by Langin et al. in an orthotopic pig-to-baboon heart xenotransplant model, machine perfusion represents the most practical and immediately applicable method for mitigating ischemia/reperfusion injury [205]. Recently, the use of NMP has been explored to advance xenotransplantation research; the results have shown that perfused organs maintained stable flow, physiological oxygenation, and largely intact architecture, as confirmed histologically [206]. Additionally, strategies such as co-stimulatory blockade with anti-CD40 and anti-CD20 antibodies, B-cell depletion in pig-to-baboon heart transplants, and the use of anti-CD83 antibodies to selectively target activated B-cells and dendritic cells have all demonstrated prolonged xenograft survival [207]. However, while the combination of these strategies may provide new opportunities for xenotransplantation, further research and interdisciplinary collaboration are needed to advance the field. (Table 2)

## 7. Summary and Future Implications

Ischemia/reperfusion injury represents one of the most common causes of vascular rarefaction and AKI immediately after kidney transplantation because of the interrelated connection between the allograft epithelium, endothelium, and circulating inflammatory cells, cytokines, antibodies, and pharmaceutical drugs. Moreover, it can raise the risk of rejection by increasing the immunogenicity of the graft. The subsequent immunological cascade involves both innate and adaptive immune responses, which play pivotal roles in mediating damage and continue to be one of the major challenges in the management of kidney transplanted patients, particularly in reducing rejection rates and preserving allograft function. While we are far from understanding the pathogenic mechanisms involved in these complex processes and there is a lack of studies addressing this intricate question, identifying potential therapeutic targets may lead to important advances. Currently, several therapeutic strategies have been developed, and their potential to minimize graft damage is promising, especially given the shared characteristics between IRI and allograft rejection.

## Figures and Tables

**Table 1:** Immune cell functions in IRI and kidney rejection.

Cell Type	Role in IRI	Role in Rejection	References
Macrophages	M1 amplifies inflammation M2 promotes repair and regeneration	TCMR: M1 polarization, antigen presentation, arteritis AMR: peritubular infiltration; modulate T-cell differentiation Chronic: M2 drives fibrosis and vasculopathy	[61,62,63,64,65,66,67,68]
Neutrophils	Release ROS, cytokines, proteases; form NETs	AMR: recruited by antibodies, NETs amplify injury, B-cells help via BAFF Acute: promote T-cell infiltration (CXCR2), recruit CD8+ via CCL1/2/5 Chronic: IL-17 production, trogocytosis	[69,70,71,72,73,74,75,76,77,78,79]
Natural Killer Cells	TEC injury and neutrophil recruitment	Distinct NK subsets linked to TCMR and AMR; strong evidence from transcriptomics	[80,81,82,83,84,85,86,87,88]
Complement System	DGF, TEC injury, fibrosis	Induction of T-cell responses, AMR, prothrombotic state	[89,90,91,92,93,94,95,96,97,98,99,100,101,102,103,104,105,106,107,108,109,110,111,112,113,114,115,116,117,118,119]
Dendritic Cells	Produce TNFα, upregulate MHC and costimulatory molecules	Promote Th1 and Tfh differentiation, supporting development of TCMR and AMR	[120,121,122,123,124,125,126,127,128,129,130]
T-cells	Th1 proinflammatory, recruits neutrophils/NK/macrophages Th2 anti-inflammatory Th17 fibrosis and neutrophil recruitment Tregs suppress inflammation CD8+ T-cells can promote injury	Th1 promotes alloimmunity Th2 supports Tregs Th17 is linked to TCMR severity Tregs induce tolerance, suppress effector T-cells CD8+ T-cells induce graft injury	[131,132,133,134,135,136,137,138,139,140,141,142,143,144,145,146,147,148,149,150,151,152,153,154,155,156,157,158,159,160,161]
B-cells	B1 cells respond to DAMPs B2 cells amplify inflammation and act as APCs	DSA production and development of AMR Promote T-cell activation Facilitate T-cell infiltration and cytokine production Chronic allograft vasculopathy and microvascular injury	[161,162,163,164,165,166,167,168,169,170,171,172,173,174,175,176,177,178,179]

**Table 2:** Treatment strategies for controlling immunological consequences of ischemia/reperfusion injury.

Strategy	Purpose	Key Features	References
Machine Perfusion	Preventive	Reduced incidence of DGF, acute rejection, and graft failure	[180]
Normothermic Regional Perfusion	Preventive	Safe alternative to in situ cooling and rapid procurement for kidneys from DCD donors	[181,182,183]
Ischemic Preconditioning	Preventive	Protected renal cells by reducing ROS, enhancing blood flow, inducing anti-apoptotic, and lowering pro-inflammatory cytokines	[184,185,186,187]
Adoptive Tregs Transfer	Therapeutic	Reduced the infiltration of immune cells	[188,189]
Immunosuppression	Therapeutic	Immunomodulation	[190,191,192,193]
B-cell-depleting Therapies	Therapeutic	May reduce IRI-related damage and rejection rates	[194,195,196,197]
Complement Inhibitors	Therapeutic	Immunomodulation	[198,199,200,201,202]
Xenotransplantation	Preventive-Therapeutic	May enhance graft viability and mitigate immune rejection	[203,204,205,206,207]

## Data Availability

No new data were created or analyzed in this study. Data sharing is not applicable to this article.

## Abbreviations

Abbreviation	Definition
AKI	Acute kidney injury
AMR	Antibody-mediated rejection
AP	Alternative pathway of the complement system
APC	Antigen-presenting cell
ATP	Adenosine triphosphate
BCR	B-cell receptor
CP	Classical pathway of the complement system
CTL	Cytotoxic T-cell
DAMP	Damaged-associated molecular pattern
DC	Dendritic cell
DGF	Delayed graft function
DSA	Donor-specific antibody
EC	Endothelial cell
FAO	Fatty acid oxidation
FB	Factor B of the complement system
FOXP3	Forkhead Box P3
HIF-1α	Hypoxia inducible factor 1α
HLA	Human leukocyte antigen
ICAM-1	Intercellular adhesion molecule-1
INF-γ	Interferon-γ
IPC	Ischemic preconditioning
IRI	Ischemia/reperfusion Injury
LP	Lectin pathway of the complement system
MAC	Membrane attack complex
NET	Neutrophil extracellular traps
NF-kB	Nuclear factor kappa B
NK	Natural killer cell
OXPHOS	Oxidative phosphorylation
REC	Renal endothelial cell
ROS	Reactive oxygen species
TCMR	T-cell-mediated rejection
TCR	T-cell receptor
TEC	Tubular epithelial cell
TGFβ	Transforming growth factor β
Th1	T helper 1 cell
Th17	T helper 17 cell
Th2	T helper 2 cell
TLRs	Toll-like receptors
Tregs	Regulatory T-cells
VCAM	Vascular cell-adhesion molecule 1
